# Trends in orphan drug use and spending among children and adolescents during 2010–2020 in Korea

**DOI:** 10.3389/fphar.2022.964426

**Published:** 2022-08-31

**Authors:** Jung Yeon Oh, Jin Yong Lee, Dong-Sook Kim

**Affiliations:** Department of Research, Health Insurance Review & Assessment Service, Wonju, South Korea

**Keywords:** orphan drugs, spending, children, adolescents, designation

## Abstract

**Background:** Since 2014 in Korea, the Ministry of Food and Drug Safety has designated orphan drugs (ODs) for the treatment of rare diseases. This study investigated the market share and 20 most frequently prescribed therapeutic subgroups of ODs among children and adolescents in Korea.

**Methods:** This study referenced the Korean national health insurance database from January 2010 through December 2020. The outcome measures were the number of prescriptions and expenditures on ODs.

**Results:** Among children aged 0–12 years, the number of patients using ODs increased from 11,264 in 2010 to 14,017 in 2020. Expenditures on ODs and their proportion of total pharmaceutical expenditures also tripled from 13.3 million USD (1.2%) in 2010 to 46.4 million USD (6.2%) in 2020. Among the overall population and adolescents aged 13–17 years, the percentage of total pharmaceutical expenditures for ODs increased from 0.4% in 2010 to 3.2% in 2020 and from 2.1% in 2010 to 11.2% in 2020, respectively. The highest numbers and drug costs of child patients were for H01 (pituitary and hypothalamic hormones and analogues, 44,839) and A16 (other alimentary tract and metabolism products, 160 million USD). The individual ODs with the highest drug costs were A16AB09 (idursulfase, 82.4 million USD) and M09AX07 (nusinersen, 36.2 million USD).

**Conclusion:** Although the market size of ODs remained small in Korea, both the number of approved ODs and the proportion of total pharmaceutical expenditures for ODs have increased. Additional policies related to designation and reimbursement should be put in place to ensure timely access to ODs.

## Introduction

Rare diseases are defined as diseases that affect a small number of people compared to the general population, and specific issues relate to their rarity. In Europe, a disease is considered to be rare when it affects 1 person per 2000 (ORPHANET) and in the United States. A rare disease is defined as a disease or condition that affects fewer than 200,000 people based on the Orphan Drug Act (FDA, 2022). According to the National Program on Rare and Intractable Diseases in Japan, rare diseases are defined as those that affect fewer than 50,000 people, or one in 2,500 (Hayashi and Umeda, 2008), and the Rare Disease Management Act in Korea specifics that rare diseases are those that affect fewer than 20,000 people (MoHw, 2019).

Orphan drugs are intended to treat diseases so rare that sponsors are reluctant to develop them under usual marketing conditions. This definition is similar in the United States, EU, and Japan. Recent continual improvements to health technology, including the introduction of innovative new drugs, have enabled the treatment of previously intractable rare diseases (Tambuyzer et al., 2020). However, treatments for rare diseases are likely to be expensive, as the number of patients is small and clinical trials are difficult (Lilford et al., 1995).

In most advanced countries, laws for orphan drugs (ODs) have provided rules for the rescue and prioritization of ODs as part of public health policies (Gammie et al., 2015), and regulations for orphan drug designations have been implemented (Micallef and Blin, 2020). The EU’s orphan designation program was launched to encourage companies to research and develop medicines for rare diseases in 2010. By the end of 2017, over 1,900 medicines had been granted orphan status and over 140 orphan medicines were marketed in the EU, providing new treatment options for patients (Ema). In the US, the number of ODs increased from 143 in 2016 to 459 in 2017, and the proportion of total pharmaceutical expenditures on ODs was 24.9% in 2017 as a result of the Orphan Drug Act of 1983 (Divino et al., 2016a; Chua and Conti, 2020). In Canada, ODs accounted for 3.3–5.6% of total pharmaceutical expenditures (Divino et al., 2016b). Other literature reviews have reported that the expenditure shares for orphan medicines within total pharmaceutical spending were frequently below 3% (Gombocz and Vogler, 2020), and that the percentage of total drug spending for orphan drugs in 2007 was 1.7% combined for France, Germany, Italy, Spain, and the UK (Orofino et al., 2010).

In Korea, the Ministry of Food and Drug Safety (MFDS) enacted regulations for the designation of ODs under the Pharmaceutical Affairs Law in 1989 and revised regulations in 2014. The MFDS has designated ODs since 2014 to encourage access to these medicines and promote their approval. Designation as an OD enables a drug to be exempted from a reexamination of new drug approval or to be prioritized for an extra process in reimbursement appraisal in the National Health Insurance (NHI) benefit scheme by the Health Insurance Review and Assessment Service. If an orphan drug passes the Pharmacopeia Committee under MFDS, it is possible to apply for the emergency drug reimbursement procedure without domestic approval, immediately after an approval review decision, including rules for the safety of medicines. Furthermore, the Korean Orphan and Essential Drug Center (KOEDC) directly imports ODs and other drugs for the treatment of patients with rare diseases.

As of December 2020, 284 ODs had been designated covering 232 total substances, some with more than one type of administration. In 2007, a positive listing system for new drugs was introduced, whereby the normal requirement for pharmaceutical companies to submit economic evaluation results for new drugs (Lee et al., 2021) would be waived for drugs treating rare diseases. South Korea achieved universal health insurance coverage through the NHI program in 1989. To reduce the burden on patients, co-payments for rare diseases (about 150 diseases) are 10%, while the general co-payments have been 20–30% since 2009. Additionally, for those who earn less than 120% of the median income, up to 50% of uncovered treatment costs are paid to reduce the burden on household finances.

However, specific OD laws have not yet been enacted in South Korea. As drug costs are expected to increase significantly due to an aging population and the emergence of new and more expensive drugs, research on efficient drug expenditures and access to medicines is necessary. To date, there are few large-scale analyses of OD use in children from South Korea. There are currently only two studies (Lee et al., 2020; Lee, 2021) on the status of ODs and OD usage specifically among children in Korea. This study, therefore, examined the use of and expenditures on ODs among children (≤12 years) and adolescents, as well as the top 20 most frequently prescribed drugs, using health insurance data from South Korea.

## Methods

### Data source and study population

This was an observational study using 2010 through 2020 health insurance data, including all inpatient and outpatient treatment records. South Korea has a single-payer health insurance system, which provides coverage for all citizens and reimburses providers on a fee-for-service basis. Demographic characteristics, medical conditions, medical service utilization (visit dates, examinations, and operations), and pharmaceutical drugs (ingredient names, number of treatment days, and drug costs) are all included in medical claims, and all claims are electronically submitted. Medical conditions are classified according to the International Classification of Diseases, 10th Revision (ICD-10). Medicines are recorded using the international nonproprietary names and codes of individual products. In South Korea, people tend to visit medical institutions to obtain prescriptions rather than purchasing over-the-counter medications at pharmacies, so most medicine use is recorded through electronic claims in the health insurance database.

We included patients eligible for National Health Insurance, Medical Aid, or the Veterans Health Service who received inpatient and outpatient medication prescriptions from tertiary hospitals, general hospitals, hospitals, or clinics from January 2010 through December 2020. For drug costs, we took a social perspective and defined drug costs as the total costs, including patient out-of-pocket costs and the value-added tax on the retail price.

### Classification of ODs

Between 2010 and 2020, a total of 185,569 products and 6,813 ingredients, doses, and formulations were listed in the market. According to the MFDS, there were 284 designated ODs covering 232 main active ingredients in December 2020, with an additional 30 ODs in the development stage.

The number of patients using certain drugs was defined as the number of patients prescribed a medication from a given therapeutic subgroup in a given year. This prevented double-counting within the same therapeutic group, but allowed counting across different therapeutic groups if a patient was prescribed drugs from multiple subgroups.

### Study measures and analysis

The study measures were the number of claims, drug expenditures, and proportion of ODs to total pharmaceutical costs. The total number of prescriptions and drug expenditures included all medicines prescribed. The analytical dimensions were age groups and drug classifications. The age groups were under 1-year, 1–6 years, 7–12 years, and 13–17 years, which were compared with adults and the elderly.

Therapeutic subgroups were classified according to the ATC classification system of the World Health Organization Collaborating Center (WHOCC, 2020). The drug classifications were based on the Anatomical Therapeutic Chemical (ATC) 2-level (therapeutic class) classifications, and off-label use was not considered. The analytical unit of this study was drugs; we analyzed each drug and performed a summation according to the ATC 2-level. The top 20 most frequently prescribed therapeutic subgroups based on the ATC 2-level classification and the main active ingredient were analyzed.

SAS Enterprise version 7.1 (SAS Institute, Cary, NC, United States) was used for all analyses.

## Results

### Approval and reimbursement of ODs

As shown in Table 1 main active ingredients were designated, of which 177 ingredients (76.3%) were approved and 158 ingredients (68.1%) reimbursed. OD designations that had been deleted or withdrawn as of 31 December 2020 were excluded. Ingredient codes of allergen extracts, house dust mites (V01) and tests for allergic diseases (V04) were also difficult to distinguish, which may have introduced some variability in the calculations.

**TABLE 1:** The number of designated, approved, and reimbursed OD products in South Korea.

ATC level-2		Designation		Approval			Reimbursement		
		No. of substances		No. of substances		No. of products	No. of substances		No. of products
Total		232		177	76.3	343	158	68.1	267
A16	Other alimentary tract and metabolism products	26	(11.2)	23	(13.0)	33	23	(14.6)	35
B01	Antithrombotic agents	7	(3.0)	5	(2.8)	10	6	(3.8)	11
B02	Antihemorrhagics	7	(3.0)	7	(4.0)	17	8	(5.1)	18
B05	Blood substitutes and perfusion solutions	1	(0.4)	2	(1.1)	4	0	(0.0)	0
B06	Other hematological agents	2	(0.9)	2	(1.1)	2	1	(0.6)	1
C01	Cardiac therapy	1	(0.4)	1	(0.6)	2	1	(0.6)	2
C02	Antihypertensives	1	(0.4)	1	(0.6)	5	0	(0.0)	0
C04	Peripheral vasodilators	1	(0.4)	1	(0.6)	0	1	(0.6)	1
C07	Beta blocking agents	1	(0.4)	1	(0.6)	1	0	(0.0)	0
C10	Lipid modifying agents	2	(0.9)	1	(0.6)	2	1	(0.6)	1
D03	Preparations for treatment of wounds and ulcers	3	(1.3)	3	(1.7)	3	1	(0.6)	1
D05	Antipsoriatics	1	(0.4)	0	(0.0)	0	0	(0.0)	0
D07	Corticosteroids, dermatological preparations	1	(0.4)	1	(0.6)	0	0	(0.0)	0
G04	Urologicals	2	(0.9)	1	(0.6)	4	1	(0.6)	4
H01	Pituitary and hypothalamic hormones and analogues	5	(2.2)	5	(2.8)	14	5	(3.2)	14
J01	Antibacterials for systemic use	2	(0.9)	0	(0.0)	0	2	(1.3)	3
J04	Antimycobacterials	2	(0.9)	2	(1.1)	2	2	(1.3)	2
J05	Antivirals for systemic use	18	(7.8)	13	(7.3)	23	12	(7.6)	20
J06	Immune sera and immunoglobulins	4	(1.7)	1	(0.6)	3	2	(1.3)	4
J07	Vaccines	2	(0.9)	0	(0.0)	0	1	(0.6)	1
L01	Antineoplastic agents	65	(28.0)	37	(20.9)	64	36	(22.8)	64
L02	Endocrine therapy	2	(0.9)	2	(1.1)	2	2	(1.3)	2
L03	Immunostimulants	10	(4.3)	8	(4.5)	17	8	(5.1)	13
L04	Immunosuppressants	17	(7.3)	14	(7.9)	31	15	(9.5)	32
M01	Antiinflammatory and antirheumatic products	1	(0.4)	1	(0.6)	1	1	(0.6)	1
M03^a^	Muscle relaxants	1	(0.4)	0	(0.0)	0	1	(0.6)	2
M05	Drugs for treatment of bone diseases	1	(0.4)	2	(1.1)	4	1	(0.6)	1
M09	Other drugs for disorders of the musculo-skeletal system	3	(1.3)	3	(1.7)	5	2	(1.3)	1
N02	Analgesics	1	(0.4)	1	(0.6)	2	0	(0.0)	0
N03	Antiepileptics	3	(1.3)	1	(0.6)	3	3	(1.9)	5
N04	Anti-parkinson drugs	1	(0.4)	0	(0.0)	0	0	(0.0)	0
N06	Psychoanaleptics	1	(0.4)	1	(0.6)	1	0	(0.0)	0
N07	Other nervous system drugs	6	(2.6)	4	(2.3)	7	3	(1.9)	4
P01	Antiprotozoals	2	(0.9)	0	(0.0)	0	2	(1.3)	2
R03	Drugs for obstructive airway diseases	3	(1.3)	3	(1.7)	3	0	(0.0)	0
R07	Other respiratory system products	4	(1.7)	6	(3.4)	9	4	(2.5)	6
S01	Ophthalmologicals	2	(0.9)	1	(0.6)	1	1	(0.6)	1
V01	Allergens	2	(0.9)	2	(1.1)	44	1	(0.6)	1
V03	All other therapeutic products	10	(4.3)	12	(6.8)	10	6	(3.8)	6
V04	Diagnostic agents	4	(1.7)	5	(2.8)	10	5	(3.2)	8
V10	Therapeutic radiopharmaceuticals	4	(1.7)	4	(2.3)	4	0	(0.0)	0

a Dantrolene sodium was withdrawn from approval, but has been reimbursed for emergency use.

By therapeutic class, the proportions of the main active ingredients of ODs were highest for L01 (antineoplastic agents), which accounted for 28% of all designated ODs, 20.9% of approved ODs, and 22.82% of drugs reimbursed through the NHI benefit schemes. The next highest proportions were for A16 (other alimentary tract and metabolism products), which accounted for 11.6% of designated ODs, 13% of approved ODs, and 14.6% of reimbursed ODs.

### Overall market share of ODs

**FIGURE 1:**
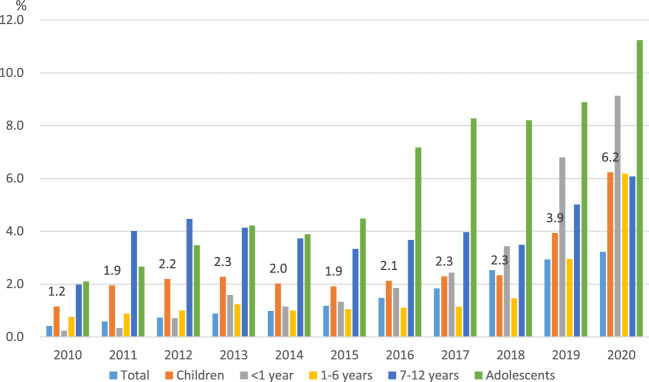
| The spending on orphan drugs as a proportion of total pharmaceutical expenditures by year.

As shown in Figure 1, the percentage of total pharmaceutical expenditures for ODs in the overall population increased from 0.4% in 2010 to 3.2% in 2020. Among children aged 0–12 years, the proportion of ODs increased from 1.2% in 2010 to 6.2% in 2020, and among adolescents aged 13–17 years from 2.1% in 2010 to 11.2% in 2020.

Table 2 summarizes the number of patients and prescriptions, as well as drug costs. The analysis of market share showed that both the volume and value of ODs steadily increased. The number of overall patients prescribed ODs increased by 3 times from 101,236 in 2010 to 310,233 in 2020, accounting for 0.6% of the total population of 51.8 million people. In children, the number of patients prescribed ODs increased from 11,264 in 2010 to 14,017 in 2013, and then decreased and remained at 11,076 in 2020. Among adolescents, the number of prescriptions for ODs increased from 1,967 in 2010 to 2,043 in 2012, and then fluctuated.

**TABLE 2:** Trends in the number of prescriptions and pharmaceutical expenditure of ODs by year.

	2010	2011	2012	2013	2014	2015	2016	2017	2018	2019	2020
No. of patients											
Total	101,236	123,464	151,401	172,085	197,255	196,333	205,587	224,317	257,405	291,300	310,233
Children	11,264	11,566	12,953	14,017	13,299	11,412	11,422	11,737	11,853	12,676	11,076
<1 year	342	329	1,466	2,601	2,817	1,551	2,523	3,302	3,289	4,266	4,306
1–6 years	5,085	5,206	5,548	5,759	5,240	5,693	5,065	4,605	4,874	4,710	3,731
7–12 years	5,837	6,031	5,939	5,657	5,242	4,168	3,834	3,830	3,690	3,700	3,039
Adolescents	1,967	2,033	2,043	1,942	1,743	1,623	1,527	1,495	1,396	1,392	1,468
No. of prescriptions (thousands)											
Total	555	725	939	1,069	1,222	1,308	1,403	1,521	1,737	2,009	2,185
Children	61	65	73	74	67	56	56	55	55	55	48
<1 year	1	1	2	4	4	2	3	4	4	5	6
1–6 years	22	23	28	29	25	24	23	22	23	22	20
7–12 years	39	41	44	41	38	30	29	29	28	28	23
Adolescents	10	12	13	13	12	11	11	11	10	11	10
Spending (million USD)											
Total	81.9	120.6	144.8	177.6	205.9	259.5	359.7	475.6	710.2	893.6	1,014.5
Children	13.3	22.3	22.4	21.3	19.4	18.3	22.7	23.0	25.6	46.0	46.4
<1 year	0.2	0.3	0.5	1.1	0.8	0.6	1.0	1.1	1.1	2.2	2.4
1–6 years	5.0	5.8	5.9	6.7	5.4	5.9	6.7	6.5	9.1	18.9	20.9
7–12 years	8.0	16.2	16.0	13.5	13.2	11.9	15.0	15.4	15.4	24.9	23.2
Adolescents	6.7	8.2	9.8	11.6	10.9	12.4	21.5	24.0	23.7	26.3	27.9

The expenditure on ODs among the overall population also increased by more than 10 times, from 81.9 million USD in 2010 to 1 billion USD in 2020. Among children, while the number of patients prescribed ODs remained about the same, the OD costs more than tripled from 13.3 million USD in 2010 to 46.4 million USD in 2020. In adolescents, the OD costs increased by around 1.5 times from 6.7 million USD in 2010 to 27.9 million USD in 2020.

For the younger age groups, OD prescriptions for patients under 1 year old increased and those for patients 1–6 years and those for patients 7–12 years decreased. The expenditures on ODs, however, increased for all three groups (Table 2).

### The most frequently prescribed ODs by therapeutic subgroup

Table 3 shows the number of prescribed patients and drug costs classified by ATC level 2. The highest number of patients was found for H01 (pituitary and hypothalamic hormones and analogues) among both children and adolescents (44,839 and 5,326, respectively).

**TABLE 3:** The number of patients using orphan drugs (ODs) and expenditures on ODs by ATC level-2.

ATC level-2		Total			Children												Adolescents (13–17 years)		
					Sum of children			<1 year			1–6 years			7–12 years					
		No. of patients (thousands)	No. of prx (thousands)	Spending (million USD)	No. of patients (thousands)	No. of prx (thousands)	Spending (million USD)	No. of patients (thousands)	No. of prx (thousands)	Spending (million USD)	No. of patients (thousands)	No. of prx (thousands)	Spending (million USD)	No. of patients (thousands)	No. of prx (thousands)	Spending (million USD)	No. of patients (thousands)	No. of prx (thousands)	Spending (million USD)
A16	Other alimentary tract and metabolism products	3	114	752.1	1	33	160.3	0	1	1.0	0	12	44.6	1	20	114.7	1	17	128.1
B01	Antithrombotic agents	46	58	98.1	2	3	5.2	1	2	0.7	0	1	2.6	0	0	1.8	0	0	2.6
B02	Antihemorrhagics	63	118	318.4	1	7	31.9	0	0	0.1	1	3	13.5	1	3	18.4	1	3	25.5
B06	Other hematological agents	0	0	0.3	0	0	0	—	—	—	—	—	—	0	0	0.0	0	0	0.0
C01	Cardiac therapy	5	53	11.4	1	3	0	1	1	0.0	0	2	0.1	0	1	0.1	0	1	0.1
C04	Peripheral vasodilators	2	5	0.4	0	0	0	0	0	0.0	0	0	0.0	0	0	0.0	0	0	0.0
C10	Lipid modifying agents	1	4	2.0	—	—	—	—	—	—	—	—	—	—	—	—	—	—	—
D03	Preparations for treatment of wounds and ulcers	11	24	8.7	0	0	0	0	0	0.0	0	0	0.0	0	0	0.0	0	0	0.0
D07	Corticosteroids, dermatological preparations	0	0	0.0	0	0	0	0	0	0.0	0	0	0.0	0	0	0.0	—	—	—
G04	Urologicals	115	1,017	141.8	0	0	0	—	—	—	0	0	0.0	—	—	—	0	1	0.1
H01	Pituitary and hypothalamic hormones and analogues	759	11,521	379.2	45	433	14.9	0	1	0.0	17	127	3.5	28	306	11.4	5	66	4.9
J01	Antibacterials for systemic use	106	274	2.5	4	7	0	1	1	0.0	2	3	0.0	1	4	0.0	2	7	0.0
J04	Antimycobacterials	1	13	44.0	0	0	0	-	-	-	0	0	0.0	-	-	-	0	0	0.1
J05	Antivirals for systemic use	43	257	255.8	2	12	4.0	1	1	0.8	1	7	1.9	1	4	1.4	1	4	1.7
J06	Immune sera and immunoglobulins	12	22	7.0	1	1	0.4	0	0	0.0	0	0	0.2	0	0	0.2	0	0	0.3
J07	Vaccines	6	17	1.3	1	2	0.1	0	0	0.0	0	1	0.1	0	1	0.1	0	0	0.0
L01	Antineoplastic agents	51	540	1,443.3	4	23	12.3	0	0	0.2	2	13	5.6	2	10	6.5	1	8	6.4
L02	Endocrine therapy	7	83	197.4	—	—	—	—	—	—	—	—	—	—	—	—	—	—	—
L03	Immunostimulants	2	20	56.6	0	0	0.2	-	-	-	0	0	0.0	0	0	0.2	0	0	0.5
L04	Immunosuppressants	40	348	630.5	1	2	4.1	0	0	0.0	1	1	1.8	0	1	2.3	1	1	3.3
M01	Antiinflammatory and antirheumatic products	6	6	2.9	5	6	2.9	5	6	2.9	0	0	0.0	0	0	0.0	0	0	0.0
M03	Muscle relaxants	0	0	0.2	0	0	0	0	0	0.0	0	0	0.0	0	0	0.0	0	0	0.0
M05	Drugs for treatment of bone diseases	4	24	0.7	0	0	0	—	—	—	—	—	—	0	0	0.0	0	0	0.0
M09	Other drugs for disorders of the musculo-skeletal system	0	1	66.4	0	0	36.2	0	0	1.7	0	0	18.5	0	0	16.0	0	0	9.2
N03	Antiepileptics	2	76	20.8	2	31	9.1	0	0	0.0	1	12	3.3	1	19	5.8	1	15	4.5
N07	Other nervous system drugs	1	12	8.9	0	0	0	—	—	—	0	0	0.0	0	0	0.0	0	0	0.0
P01	Antiprotozoals	0	0	0.0	0	0	0	0	0	0.0	—	—	—	—	—	—	—	—	—
R07	Drugs for obstructive airway diseases	22	29	4.5	21	27	3.7	20	26	3.6	1	1	0.1	0	0	0.0	0	0	0.0
S01	Ophthalmologicals	1	2	0.8	0	0	0	-	-	-	-	-	-	0	0	0.0	0	0	0.0
V01	Allergens	0	0	0.0	0	0	0	-	-	-	-	-	-	0	0	0.0	-	-	-
V03	All other therapeutic products	16	51	16.0	1	3	2.5	0	1	0.2	1	2	1.6	0	1	0.7	0	1	0.7
V04	Diagnostic agents	58	116	56.0	0	0	0	-	-	-	0	0	0.0	0	0	0.0	0	0	0.1

prx, prescriptions.

In the overall population, the highest spending was found for L01 (antineoplastic agents, 1.4 billion USD) and A16 (other alimentary tract and metabolism products, 0.7 billion USD). Among children, the highest spending was found for A16 (other alimentary tract and metabolism products, 160 million USD), and M09 (other drugs for disorders of the musculoskeletal system, 36 million USD). Among adolescents, the highest spending was on A16 (other alimentary tract and metabolism products, 128.1 million USD) and B02 (antihemorrhagics, 25.5 million USD).

By age group, for under 1-year-old children the highest spending was for R07 (other respiratory system products, 3.6 million USD), and for those 1–6 years and 7–12 years of age it was A16 (other alimentary tract and metabolism products, 44.6 million USD and 114.7 million USD respectively).

Table 4 shows the results by the main active substance. Among children the highest spending was for A16AB09 (idursulfase, 82.4 million USD), M09AX07 (nusinersen, 36.2 million USD) B02BD08 (coagulation factor, 23.8 million USD), and A16AB07 (alglucosidase alfa, 16.3 million USD). In adolescents, the highest spending was on A16AB09 (idursulfase, 54.5 million USD), and B02BD08 (coagulation factor, 25.1 million USD).

**TABLE 4:** Top 20 main active ingredients for orphan drugs by spending level.

	Children		Adolescents				Overall population					
	atc_code	atc_name	No. of prx (thousands)	Spending (million USD)	atc_code	atc_name	No. of prx (thousands)	Spending (million USD)	atc_code	atc_name	No. of prx (thousands)	Spending (million USD)
1	A16AB09	idursulfase	13	82.4	A16AB09	idursulfase	7	54.5	L01EB04	osimertinib	55	447.7
2	M09AX07	nusinersen	0	36.2	B02BD08	coagulation factor VIIa	2	25.1	H01BA02	desmopressin	11,356	336.6
3	B02BD08	coagulation factor VIIa	3	23.8	A16AB02	imiglucerase	1	16.6	B02BD08	coagulation factor VIIa	18	261.5
4	A16AB07	alglucosidase alfa	3	16.3	A16AB12	elosulfase alfa	1	13.1	L04AX05	pirfenidone	241	222.2
5	A16AB02	imiglucerase	2	15.2	A16AB07	alglucosidase alfa	1	11.9	L01ED01	crizotinib	35	215.5
6	H01BA02	desmopressin	433	14.9	A16AB05	laronidase	1	11.5	L04AA25	eculizumab	14	205.1
7	A16AX07	sapropterin	3	11.5	M09AX07	nusinersen	0	9.2	A16AB09	idursulfase	26	199.0
8	A16AB05	laronidase	3	11.1	A16AB04	agalsidase beta	1	9.0	A16AB04	agalsidase beta	22	171.4
9	A16AB12	elosulfase alfa	1	10.6	H01BA02	desmopressin	66	4.9	L02BB04	enzalutamide	60	170.4
10	B02BD06	von Willebrand factor and coagulation factor VIII in combination	2	7.9	A16AB08	galsulfase	0	4.2	G04BX15	pentosan polysulfate sodium	1,017	141.8
11	N03AX17	stiripentol	6	6.8	A16AX07	sapropterin	0	3.5	L01ED03	alectinib	26	120.1
12	A16AB08	galsulfase	0	4.3	L04AA04	antithymocyte immunoglobulin (rabbit)	1	2.6	J05AR18	emtricitabine, tenofovir alafenamide, elvitegravir and cobicistat	59	110.4
13	L01AC01	thiotepa	1	4.1	N03AX17	stiripentol	1	2.4	A16AB02	imiglucerase	8	103.0
14	A16AX03	sodium phenylbutyrate	2	3.8	N03AF03	rufinamide	14	2.1	L01XG02	carfilzomib	80	86.1
15	R07AB	caffeine anhydrous + citric acid	27	3.7	L01AC01	thiotepa	0	1.9	L01XC17	nivolumab	37	83.4
16	J05AB06	ganciclovir	9	2.9	B01AX01	defibrotide	0	1.8	L01BC08	decitabine	100	83.1
17	M01AE01	ibuprofen	6	2.9	L01BB06	clofarabine	0	1.3	L04AA04	antithymocyte immunoglobulin (rabbit)	18	77.5
18	B01AX01	defibrotide	0	2.8	A16AX12	trientine	2	1.3	A16AB07	alglucosidase alfa	8	76.8
19	L01XX02	asparaginase	20	2.6	L01XX02	asparaginase	7	1.2	M09AX07	nusinersen	1	66.4
20	L01XC19	blinatumomab	0	2.4	A16AX03	sodium phenylbutyrate	0	1.1	L01EL01	Ibrutinib	10	63.6

prx, prescriptions.

## Discussion

The Korean government regulated the designation of ODs under the Pharmaceutical Affairs Law in 1989 and established the OD Center in 1999. ODs have been designated since 2014, and as of December 2020, 232 ODs had been designated, with 177 ingredients among them approved (76.3%), and 158 ingredients reimbursed (68.1%).

The current study showed that the market size of ODs and OD costs as a proportion of total drug costs in the overall population and children increased from 81.9 million USD (0.4%) and 13.3 million USD (1.2%) in 2010 to 1 billion USD (3.2%) and 46.4 million (6.2%) in 2020. The ratio of ODs costs to total drug costs was higher (6.2%) for children than for adults (3.2%) in 2020, which is consistent with a previous study that found that the cost per patient for ODs in the United States in 2018 was higher in children at 5,467 USD compared to adults at 3,654 USD (Chua and Conti, 2020). The proportion of total health care costs accounted for by ODs, however, increased only from 4 to 6.6% between 2013 and 2018 in children aged 0–17 years compared to an increase of 5.6–9.2% in adults aged 18–64 years (Chua and Conti, 2020).

In Europe, the share of the total pharmaceutical market represented by ODs increased from 3.3% in 2010 to a peak of 4.6% in 2016 after which it has been predicted to level off through 2020, as growth would fall in line with that in the wider pharmaceutical market (Mestre-Ferrandiz et al., 2019). In Canada, expenditures on 147 ODs totaled $610.2 million in 2007 and $1,100.0 million in 2013, representing 3.3 and 5.6% of total Canadian pharmaceutical drug expenditures in 2007 and 2013, respectively.

This study also shows that the cost by therapeutic class in children was the highest for A16 (other alimentary tract and metabolism products, 160.3 million USD), and M09 (other drugs for disorders of the musculoskeletal system, 36.2 million USD). In adolescents, A16 (other alimentary tract and metabolism products, 128.1 million USD) and B02 (antihemorrhagics, 25.5 million USD) were the highest. There was no previous study on the market size for each component of orphan drugs, so a direct comparison was not possible.

Most European countries have not implemented pricing and reimbursement policies specific to ODs and the availability of ODs varies between countries (Sarnola et al., 2018). They have, however, continuously raised the need for incentives to authorize innovative treatments for rare diseases (Micallef and Blin, 2020; Aartsma-Rus et al., 2021), while recognizing that the high price of ODs is likely to be a challenge to the sustainability of healthcare expenditures (Hughes-Wilson et al., 2012; Mestre-Ferrandiz et al., 2019). Policies to encourage the availability of more ODs are a positive development for patients; however, high prices are putting pressure on health care budgets and raising questions about whether those prices are appropriate (Chambers et al., 2020). The United States was the first nation to introduce orphan drug legislation with the Orphan Drug Act of 1983, which dramatically increased the approval of ODs. In the United States, there were 768 approved OD indications as of December 2018, which represented 526 individual ODs, as shown by Chua et al. (2021) (Chua and Conti, 2020). As of October 2010, 720 drugs had received OD designations from the European Medicines Agency (EMA) through October 2010 (Schey et al., 2011). In Korea, numerous drugs in the metabolism, antineoplastic agent, and immunosuppressant classes have been listed, and drug costs in the metabolism and antihemorrhagic classes have been high, despite differences in the prevalence of rare diseases.

To our knowledge, this is the first study to describe the national market share trend of ODs in children relative to adolescents and the total population, as well as the top 20 frequently prescribed therapeutic subgroups from 2010–2020 in Korea. Using a very large dataset (i.e., the total population) means that the results of this study both cover rare diseases and can be generalized for epidemiological analyses. In addition, the NHI claims data includes all healthcare service utilization and medications in hospitals and clinics, indicating that the results of this study can suggest meaningful trends for ODs.

This study has the following main limitations. First, direct comparisons of the study findings are difficult due to differences in both the prevalence of rare diseases (due to racial issues, etc.) and the main active ingredients of designated ODs. Second, our dataset covers the entire national population including foreigners in Korea; clearly, patient characteristics in other countries may differ from this sample. For example, the number of encounters with physicians and the number of drug companies is relatively high in Korea. Third, only 68.1% of designated ODs were reimbursed, and some variation might have been introduced into these results if some prescriptions not covered by the NHI were included in the claims data. Fourth, this study analyzed an open cohort for the 10-year period; as children grew up, transitions from one age group to another could occur. However, the main purpose of the study was to compare patterns by year, and the shift of age groups is an unavoidable limitation.

This is the first study to investigate trends in ODs among both the entire population and children in Korea. To promote access to ODs in children, special attention and further studies should be considered to compare ODs between countries.

## Data Availability

The datasets presented in this article are not readily available because Data can be accessed with restriction because the analysis can be performed after the approval of our institution. Requests to access the datasets should be directed to https://opendata.hira.or.kr/home.do.
